# Quality of life of adolescents with cerebral palsy: agreement between self-report and caregiver’s report*

**DOI:** 10.1590/1518-8345.3928.3300

**Published:** 2020-07-01

**Authors:** Mariana Ceravolo Ferreira, Nathália Ribeiro Garcia, Cejane Oliveira Martins Prudente, Maysa Ferreira Martins Ribeiro

**Affiliations:** 1 Universidade Federal de Goiás, Faculdade de Medicina, Goiânia, GO, Brazil.; 2 Scholarship holder at the Coordenação de Aperfeiçoamento de Pessoal de Nível Superior (CAPES), Brazil.; 3 Pontifícia Universidade Católica de Goiás, Escola de Ciências Sociais e da Saúde, Goiânia, GO, Brazil.

**Keywords:** Quality of Life, Cerebral Palsy, Adolescent, Young Adult, Child, Caregivers, Qualidade de Vida, Paralisia Cerebral, Adolescente, Adulto Jovem, Criança, Cuidadores, Calidad de Vida, Parálisis Cerebral, Adolescente, Adulto Joven, Niño, Cuidadores

## Abstract

to assess the quality of life (QOL) of adolescents with cerebral palsy (CP) by self-report and by the caregiver’s report, and to analyze the agreement between these reports.

**Method:**

cross-sectional study conducted with 101 adolescents with CP and 101 caregivers. Both answered the *Pediatric Quality of Life Inventory* (PedsQL), module 4.0 - Generic (PedsQL 4.0) and module 3.0 - PC (PedsQL 3.0). Agreement between reports was analyzed using the *Mann-Whitney* test and the intra-class correlation coefficient (ICC) (p<0.05).

**Results:**

the lowest scores were in physical health, school activities and fatigue in the self-report. The lowest scores were in physical health and daily activities, in the caregivers’ report. Perceptions among adolescents and caregivers differed in physical health, movement and equilibrium, daily and school activities, with a lower score for caregivers in all of them. The agreement between the self-report and the caregivers’ report was poor (ICC<0.44) and in both instruments, the caregivers’ report was less optimistic.

**Conclusion:**

physical health is the most impaired domain of the QOL of adolescents with CP, both in the self-report and in the caregivers’ report. However, there is poor agreement between these reports, emphasizing that the use of the caregivers’ report should be cautious.

## Objetivo

avaliar a qualidade de vida (QV) de adolescentes com paralisia cerebral (PC) pelo autorrelato e pelo relato do cuidador, e analisar a concordância entre estes relatos.

**Método:**

estudo transversal conduzido com 101 adolescentes com PC e 101 cuidadores. Ambos responderam o *Pediatric Quality of Life Inventory* (PedsQL), módulo 4.0 - Genérico (PedsQL 4.0) e módulo 3.0 - PC (PedsQL 3.0). A concordância entre os relatos foi analisada pelo teste de *Mann-Whitney* e pelo coeficiente de correlação intra classe (ICC) (p<0.05).

**Resultados:**

no autorrelato os menores escores foram em saúde física, atividades escolares e fadiga. No relato dos cuidadores as menores pontuações foram em saúde física e atividades cotidianas. As percepções entre adolescentes e cuidadores divergiram em saúde física, movimento e equilíbrio, atividades cotidianas e escolares, com menor escore dos cuidadores em todos eles. A concordância entre o autorrelato e o relato dos cuidadores foi pobre (ICC<0.44), e em ambos instrumentos o relato dos cuidadores foi menos otimista.

**Conclusão:**

a saúde física é o domínio mais prejudicado da QV de adolescentes com PC, tanto no autorrelato quanto no relato dos cuidadores. Porém há pobre concordância entre estes relatos, ressaltando que o uso do relato dos cuidadores deve ser cauteloso.

evaluar la calidad de vida (CV) de adolescentes con parálisis cerebral (PC) a partir del autorrelato y del relato del cuidador, y analizar la concordancia entre esos relatos.

**Método:**

estudio transversal, llevado a cabo con 101 adolescentes con PC y 101 cuidadores. Ambos contestaron el *Pediatric Quality of Life Inventory* (PedsQL), módulo 4.0 - Genérico (PedsQL 4.0) e módulo 3.0 - PC (PedsQL 3.0). La concordancia entre los relatos se analiz*ó por* el test de *Mann-Whitney* e por el coeficiente de correlación intra clase (ICC) (p<0.05).

**Resultados:**

en el autorrelato, los puntajes *más bajo*s se dieron en salud física, actividades escolares y fatiga. En el relato de los cuidadores, los puntajes más bajos se dieron en salud física y actividades cotidianas. Las percepciones de los adolescentes y los cuidadores fueron divergentes en salud física, movimiento y equilibrio, actividades cotidianas y escolares, con un puntaje más bajo por parte de los cuidadores en todos esos rubros. La concordancia entre el autorrelato y el relato de los cuidadores fue pobre (ICC<0.44), y en ambos instrumentos el relato de los cuidadores fue menos optimista.

**Conclusión:**

la salud física es el dominio más perjudicado de la CV de los adolescentes con PC, tanto en el autorrelato como en el relato de los cuidadores. Sin embargo, la concordancia entre los dos relatos es deficiente, razón por la cual, el relato de los cuidadores debe realizarse con cautela.

## Introduction

Cerebral palsy is the main cause of physical disability in childhood, with a prevalence of 2 to 2.5 cases *per* 1000 live births in countries^(^ 1 ^)^. It is a chronic condition resulting from brain injury in the first years of life that causes persistent impairments and limitations throughout life^(^ 2 ^)^. Changes in muscle tone and movement are the main characteristics, impairing functionality, hindering independence and interfering with the affected person’s quality of life^(^ 3 ^-^ 4 ^)^. The degree of impairment is variable, with frequent association of motor disorder with comorbidities, such as musculoskeletal deformities, sensory, cognitive, behavioral problems and seizures^(^ 2 ^)^.

In the transition to adolescence, people with cerebral palsy start to suffer due to the increase of weight and body size, evolving with functional losses. The limitations become more evident at a stage in life that imposes countless challenges. Personality disorders and the search for autonomy and independence, inherent to adolescence, face the dependence on their caregivers to perform many activities^(^ 3 ^,^ 5 ^-^ 7 ^)^.

Previous studies show the poor quality of life of this population when compared to healthy adolescent^(^ 6 ^,^ 8 ^-^ 9 ^)^, highlighting the need for research on all aspects of quality of life of adolescents with cerebral palsy, especially in low and middle income countries^(^ 9 ^-^ 11 ^)^. In addition, the limitation of centers specialized in rehabilitation in the care of adolescents with cerebral palsy is notorious. The vast majority have programs aimed at children and adults with disabilities, neglecting support for adolescents^(^ 7 ^,^ 11 ^-^ 12 ^)^.

The same happens in the research field. Most studies that assess quality of life in cerebral palsy include children and adolescents in the same sample^(^ 9 ^,^ 13 ^-^ 15 ^)^, use only one type of instrument (generic or specific)^(^ 13 ^-^ 14 ^,^ 16 ^)^, give preference to the report of caregivers instead of self-report and suggest discrepancy between self-report and caregiver’s report^(^ 6 ^,^ 8 ^,^ 13 ^,^ 15 ^,^ 17 ^)^. There are no studies conducted only with adolescents, which assess quality of life by self-report and use generic and specific instruments simultaneously. Therefore, it is essential to listen to adolescents with cerebral palsy, in order to understand their needs and favor assistance focused on increasing the quality of life of this population.

The results of this study will highlight the psychosocial and health needs of adolescents with cerebral palsy under their perception and that of their caregivers, facilitating the care directed to this population. Thus, this study aims to assess the quality of life of adolescents with cerebral palsy from the self-report and the caregiver’s report, as well as to analyze the agreement between them.

## Method

Cross-sectional study, carried out between March and August 2017, in all public and/or philanthropic educational/or rehabilitation institutions in Goiânia, Brazil. The project was approved by the ethics committee of the institution proposing the research, CAAE nº 64557517.0.0000.0037, opinion nº 1.940.559.

The search for the sample took place in all nine public and/or philanthropic educational and/or rehabilitation institutions in Goiânia. A screening was carried out in these places to identify adolescents between 10 and 19 years old with a diagnosis of cerebral palsy, obtaining a population of 236 individuals distributed in six co-participating institutions, of the nine consulted. Of these, adolescents who had genetic syndromes associated with cerebral palsy were excluded, and all the remaining adolescents were subjected to a standardized “test question” to assess their ability to understand and communicate. This question corresponded to the first item of the generic PedsQl 4.0 instrument (Is it difficult to put your shoes on?). Adolescents who did not understand or answer the question did not participate in the study. Thus, after applying the exclusion criteria, the sample consisted of 101 adolescents with cerebral palsy and 101 caregivers. Caregivers were considered those who lived with the adolescent, accompanied them in teaching and/or rehabilitation activities, being the main responsible for their assistance.

In this study, we chose to study only adolescents, given the peculiarities and individualities of the adolescent age group that require individualized analysis, as well as the lack of studies aimed exclusively at this population.

After defining the sample, caregivers were invited to participate in the study and to authorize the participation of adolescents by signing the Free and Informed Consent Form (FICF). The adolescents were also informed about the research and invited to take part through the Free Informed Assent Form (FIAF).

The data were collected in the participating institutions, through individual interviews, in a reserved place, lasting approximately 20 minutes. All research instruments were applied by five previously trained and calibrated researchers. Initially, caregivers answered a sociodemographic form, reporting their data (family relationship with the adolescent, age, gender, work activities and education level) and data about the adolescents (age, gender, comorbidities and school nature). Then, caregivers and adolescents answered, in separate rooms, the instrument for assessing the quality of life of adolescents, Pediatric Quality of Life Inventory (PedsQL), module 4.0 - Generic (PedsQL 4.0) and module 3.0 - Cerebral Palsy (PedsQL 3.0). At the end of the application of PedsQL to adolescents, researchers based on their clinical expertise, assessed the level of motor impairment using the Gross Motor Function Classification System (GMFCS).

PesdQL 4.0 assesses more global aspects of quality of life and consists of 23 items divided into four domains: physical function, emotional function, social function and school function. These domains can be grouped into three scores: physical health, psychosocial health (school function, social function and emotional function) and total score. PedsQL 3.0 has 35 questions distributed in seven domains: daily activities, school activities, movement and equilibrium, pain, fatigue, food and speech. The questions in the two questionnaires refer to the number of times that each item was a problem during the last month and the answers are given on a Likert scale (0 = never, 1 = almost never, 2 = sometimes, 3 = many times, 4 = almost always). The score of the domains ranges from 0 to 100, where zero is the worst score^(^ 18 ^)^. All instruments used were validated for the Portuguese language^(^ 19 ^-^ 20 ^)^.

The GMFCS classifies children and adolescents with cerebral palsy into five levels of motor skills. At level I, they walk without limitations and present only some difficulties in equilibrium and coordinating more advanced activities (running and jumping). At level II, they need a mobility device in the community and to walk long distances. At level III, they use mobility devices in the internal environment and in the community, they use mobility on wheels. At level IV, self-movement is limited, being transported in a manual wheelchair or using motorized mobility. At level V, limitations are severe, head and trunk control is precarious and self-movement is possible only with a motorized wheelchair^(^ 2 ^)^.

The data, entered by two researchers, were processed and analyzed using the Statistical Package for Social Science (SPSS), version 23.0 for Windows. The sample was described with relative and absolute frequency, mean, standard deviation (SD), median and confidence interval (95% CI). Data on quantitative variables were checked for normality using the Kolmogorov-Smirnov test and those that did not have a normal distribution were described by median in order to provide a more reliable description of the sample. Agreement between adolescents’ and caregivers’ reports was analyzed using the Mann-Whitney test and the one-way random intra-class correlation (ICC) coefficient (p < 0.05), considering a ICC value <0.50 poor agreement, 0.50 to 0.75 moderate, 0.75 to 0.90 good and ICC > 0.90 excellent^(^ 21 ^)^.

## Results

The study sample consisted of 101 adolescents with cerebral palsy and 101 caregivers. Most adolescents were male, with a mean age of 14.8 years old (± 2.9). More than half had no comorbidities, most were classified as GMFCS levels I, II and III and 91.1% attended school (Table 1). Regarding caregivers, 80.2% were mothers of adolescents, with a mean age of 42 years old (± 7.2). Most did not work (67.9%) and had schooling up to high school (90.1%).

**Table 1:** Absolute (n) and relative (%) frequency of characteristics of adolescents with cerebral palsy. Goiânia, GO, Brazil, 2017

Characteristics	Participants (n=101)	
	n	%
Gender		
Female	43	42.6
Male	58	57.4
Age group		
10 to 14 years	50	49.5
15 to 19 years old	51	50.5
Comorbidities		
Yes	44	43.6
No	57	56.4
GMFCS*		
I, II and III	78	77.2
IV and V	23	22.8
School	92	91.1
Regular	64	69.6
Special	28	30.4

* GMFCS = Gross Motor Function Classification System

In PedsQL 4.0, the physical health score was the most affected, both in the self-report of adolescents and caregivers. In PedsQL 3.0, in the adolescent’s perception, the most affected domains were school activities and fatigue; different from the caregiver’s perception, in which the domain with the lowest score was daily activities. In all domains with mean difference, both in PedsQL 4.0 and 3.0, caregivers had an underestimated perception of adolescents’ quality of life, with lower scores (Table 2).

**Table 2:** Description and comparison between the self-report of quality of life of adolescents with cerebral palsy and the report of caregivers. Goiânia, GO, Brazil, 2017

PedsQL*	Adolescent self-report Median (IQ)^†^	Caregiver's report Median (IQ)^†^	p-value
PedsQL* 4.0			
Physical health	53.1(37.5-62.5)	34.3 (21.8-53.1)	<0.01^‡^
Psychosocial health	55.0 (47.5-65.0)	53.3 (43.3-59.1)	0.04
Total score	53.2 (46.7-62.5)	46.7 (39.1-54.8)	<0.01^‡^
PedsQL* 3.0			
Daily activities	63.8 (37.5-72.2)	47.2 (18.0-63.8)	<0.01^‡^
School activities	56.2 (43.7-75.0)	50.0 (21.8-65.6)	<0.01^‡^
Movement and equilibrium	60.0 (50.0-75.0)	50.0 (45.0-65.0)	<0.01^‡^
Pain	68.7 (43.7-75.0)	68.7 (50.0-75.0)	0.27
Fatigue	56.2 (43.7-68.7)	62.5 (43.7-68.7)	0.12
Food	60.0 (45.0-75.0)	55.0 (35.0-70.0)	0.11
Speech and communication	62.5 (37.5-75.0)	56.2 (37.5-68.7)	0.50

* PedsQL = Pediatric Quality of Life Inventory;

† IQ = interquartile range;

‡ statistically significant difference after comparison using the Mann-Whitney(p<0.05)

There was poor agreement between the self-report and the caregivers’ report on the PedsQL 4.0 psychosocial health score and, except for movement and equilibrium, in all other domains of PedsQL 3.0. These data suggest that caregivers and adolescents with cerebral palsy have different perceptions of quality of life (Table 3).

**Table 3:** Agreement between the self-report of quality of life of adolescents with cerebral palsy and the report of caregivers in the PedsQL* 4.0 and PedsQL* 3.0. Goiânia, GO, Brazil, 2017

PedsQL*	ICC^†^	p-value	IC 95%^‡^
PedsQL* 4.0			
Physical health	0.19	0.03	-0.06-0.37
Psychosocial health	0.23	<0.01^§^	0.04-0.41
Total Score	0.17	0.04	-0.03-0.35
PedsQL*3.0			
Daily activities	0.44	<0.01^§^	0.27-0.58
School activities	0.33	<0.01^§^	0.14-0.49
Movement and equilibrium	0.15	0.07	-0.05-0.33
Pain	0.23	0.01	0.04-0.41
Fatigue	0.20	0.02	0.01-0.38
Food	0.37	<0.01^§^	0.19-0.53
Speech and communication	0.29	<0.01^§^	0.01-0.45

* PedsQL = Pediatric Quality of Life Inventory;

† ICC = intra-class correlation coefficient of the caregiver-adolescent interobserver reliability;

‡ CI 95% = Confidence Interval;

§ statistically significant difference (p<0.05)

## Discussion

In this study, we evaluated the quality of life of adolescents with cerebral palsy based on self-report and the caregivers’ reports, as well as analyzing the agreement between them. In the adolescents’ self-report, the lowest scores were in school activities and fatigue. In the caregivers’ report, they were in daily activities. Both caregivers and adolescents scored less on the physical health score. The agreement between the adolescents’ self-report and the caregivers’ report was poor and there was a tendency for caregivers to underestimate the adolescents’ quality of life.

In line with previous works^(^ 8 ^,^ 10 ^,^ 15 ^)^, in this study, physical health was the most impaired in PedsQL 4.0, both in the reports of adolescents and caregivers. Adolescence is a period of physical and psychological maturation^(^ 3 ^)^. Increased body size and weight, as well as musculoskeletal changes are inherent in this phase. Consequently, individuals with cerebral palsy, who already have difficulty with movement and locomotion, suffer even more from all these changes when going through adolescence^(^ 22 ^)^. Activities previously performed are now difficult to perform or are no longer performed. Thus, many evolve with muscle weakness, musculoskeletal deformities, decline in flexibility and functionality. Therefore, it is natural that the physical function of these young people decreases with advancing age^(^ 3 ^,^ 22 ^)^. Furthermore, adolescence is a phase in which “normality patterns” are more questioned and a concern with body image is common^(^ 23 ^)^. However, adolescents with cerebral palsy often use assistive locomotion devices or orthoses that are not in line with these social standards.

In PedsQL 3.0, the domains of school activities and fatigue had the worst performance in the adolescents’ point of view. In the school environment, mainly of a regular nature, there is the lack of the caregiver to help the adolescents, and a large part of the sample in this study attended such a school. Thus, there is a need to face obstacles, increasing levels of physical and mental fatigue^(^ 3 ^)^. Also, facing the difficulties contributes to the increase in the capacity to perceive their conditions and limitations in a phase in which there is a decline in function and the emergence of self-acceptance conflicts^(^ 4 ^)^. All of these factors are associated with the increase in physical and mental fatigue reported by adolescents linked to the impaired perception of school activities. Furthermore, the domain of school activities in the PedsQL 3.0 instrument assesses the difficulty in fine motor skills^(^ 18 ^)^, difficult to be performed by people affected by cerebral palsy, even more in older adolescents^(^ 22 ^)^.

On the other hand, under the caregiver’s assessment, the worst performance occurred in daily activities. In PedsQL 3.0, the domain of daily activities assesses the difficulty with clothing and hygiene^(^ 18 ^)^. It is noteworthy that these tasks are commonly performed in the home environment. In it, caregivers do most of the activities for adolescents, demanding time and generating distress. Thus, it is understandable that they have an impaired view of this domain, due to the fact that they do not let the adolescents perform the activities, because they have the idea that it is very difficult for them^(^ 24 ^)^. From another perspective, adolescents may not find these activities complicated, since they are usually performed by caregivers, requiring no effort and coping^(^ 22 ^)^. Such is that, in the self-report, the domain of daily activities had the second highest score. Thus, it is noted that caregivers find it easy to perceive what is inherent to their care, while they have difficulty visualizing the difficulties of adolescents with the external environment, in which they are not present.

In accordance with the recent literature^(^ 8 ^,^ 11 ^,^ 13 ^,^ 17 ^)^, there was a poor agreement between the adolescents’ and caregivers’ report (ICC<0.44) and mean difference in physical and total health scores (PedsQL 4.0), and in the domains of daily activities, school activities and movement and balance (PedsQL 3.0). In addition, in all scores and domains with a mean difference between the reports, caregivers had an impaired perception of the adolescents’ quality of life, with lower scores.

Previous studies^(^ 6 ^,^ 13 ^,^ 15 ^)^ show that parents of healthy adolescents tend to overestimate their children’s quality of life, and parents of adolescents with chronic health conditions tend to underestimate it. In general, caregivers of people with cerebral palsy are much more concerned and melancholic about the limitations than the adolescents themselves^(^ 22 ^)^.

Parents anchor their children’s perception of quality of life in their own experiences and expectations; and they associate the lack of pleasure in their children’s lives with the limitations and precarious opportunities to choose their preferences^(^ 12 ^)^. They also report that sexuality and the relationship with the opposite sex are important for their quality of life^(^ 3 ^)^. In addition, concerns about the scarcity of financial resources and health services available for multi-professional monitoring are mentioned in the caregivers’ reports^(^ 25 ^)^.

From another perspective, adolescents do not cite sexuality, development of relationships, access to services and financial resources as determinant aspects for a better quality of life^(^ 3 ^)^. For them, social factors such as family support, living with peers without disabilities, attending school, going out and talking with friends are decisive in the way they perceive quality of life. In addition, the development of self-defense strategies to overcome daily barriers, the ability to face difficulties and the perception and acceptance of the self, of their real conditions are also important points^(^ 4 ^)^.

The quality of life report of adolescents with cerebral palsy is also influenced by specific factors^(^ 3 ^)^. Cerebral palsy is a condition that begins in early childhood^(^ 2 ^)^ and that, with advancing age, affected individuals have already adapted to their limitations and developed strategies to overcome them. Thus, most adolescents state that cerebral palsy is something they can overcome^(^ 4 ^)^. Unlike parents, teenagers cope better with their limitations. In addition, the experiences they have are different, as well as the beliefs about disability and their well-being^(^ 15 ^,^ 17 ^)^.

Given this information, it is noted that adolescents and caregivers differ in concerns and expectations in relation to what is most important for a good quality of life. It is worth highlighting the reality of rehabilitation centers, where therapies and activities are centered on younger children and adolescents up to 12 years old, neglecting older adolescents. After that age, they are no longer a priority, discharged and have no access to almost any type of service. This may have been a determining factor for parents’ underestimated perception. In addition, it is common that, over time, parents of adolescents have no hope of prognostic evolution and this, coupled with the lack of opportunity for inclusion and expectations about a future, make them have a worse perception of children’s quality of life^(^ 12 ^)^. Thus, probably, the result of poor agreement between the reports is more a consequence of the experiences, expectations and concerns of each individual than a question of who is right or wrong^(^ 9 ^,^ 15 ^)^.

In addition to the interference of individual aspects in the results of this study, it is important to note that the way of reasoning differs from person to person. In the response to the quality of life instrument, adolescents first respond and then justify. Parents do the opposite, which can bring more influence of personal experiences and beliefs to the report. Furthermore, adolescents tend to base the answer on a single example, while parents respond based on what they think their child would say, and not on their real perception^(^ 25 ^)^.

The limitations of this study were the cross-sectional epidemiological design, a fact that prevents the identification of risk factors for poor quality of life. In addition, the restriction of the sample to adolescents without serious communication impairment and linked to an educational or rehabilitation institution requires caution in interpreting the results, and the same sample profile must be observed. Thus, it is important to reproduce longitudinal studies that also include adolescents not assisted by institutions, making it possible to expand the generalization of results. 

On the other hand, the strength of this work is given by the use of methods suggested by recent literature^(^ 10 ^-^ 11 ^)^. It covered all institutions in the municipality, included only adolescents in the sample, used an integrated instrumental approach; with generic and specific quality of life instruments for cerebral palsy; and it involved the perception of the adolescent and the caregiver. The results found highlight the needs of adolescents with paralysis under their perception and under the perception of their caregiver, suggesting that it is necessary to pay attention to the perspective of the adolescents themselves regarding their real needs. Thus, these results will contribute to broaden the understanding of the needs of adolescents with cerebral palsy and facilitate the directing of public health and assistance policies to the period of adolescence, enabling the improvement of the quality of life of this population.

## Conclusion

This study evaluated the quality of life of adolescents with cerebral palsy from two perspectives. In the view of adolescents, the most affected dimensions are physical health, school activities and fatigue, unlike the view of caregivers, in which the most compromised dimensions are physical health and daily activities. There was poor agreement between the adolescents’ self-report and the caregivers’ report, the caregivers tended to a pessimistic view of the adolescents’ quality of life. Despite this, both reports emphasize that the physical health of adolescents with cerebral palsy needs targeted attention. Thus, it is recommended to create specific programs for this age group, providing inclusion in rehabilitation and sports activities and facilitating the practice of physical exercises to control body weight, maintain muscle strength and flexibility, improving the quality of life of this population.

Finally, the more optimistic perception of adolescents suggests that the caregivers’ report is less reliable when evaluated in isolation, without the adolescent’s report. As a complement, the caregiver’s report is valid, since different views not perceived by adolescents can add to the results, not being recommended their isolated use for adolescents capable of self-reporting their quality of life. As a complement, the caregiver’s report is valid, since different views not perceived by adolescents, can add to the results, but their isolated use is not recommended for adolescents capable of self-reporting their quality of life. In addition, whenever possible, it is best to assess the quality of life of adolescents with cerebral palsy based on their own report.
